# Hemodynamic parameters in patients undergoing surgery for pheochromocytoma/paraganglioma: a retrospective study

**DOI:** 10.1186/s12957-023-03072-z

**Published:** 2023-06-27

**Authors:** Giuseppina De Filpo, Gabriele Parenti, Clotilde Sparano, Giulia Rastrelli, Elena Rapizzi, Serena Martinelli, Francesca Amore, Benedetta Badii, Prosperi Paolo, Tonino Ercolino, Massimo Mannelli, Mario Maggi, Letizia Canu

**Affiliations:** 1 Department of Experimental and Clinical Biomedical Sciences “Mario Serio”, University of Florence, Florence, Italy; 2 Endocrinologic Unit, Azienda Ospedaliera Universitaria Careggi, Florence, Italy; 3 Sexual Medicine and Andrology Unit Department of Experimental and Clinical Biomedical Sciences “Mario Serio”, University of Florence, Florence, Italy; 4 Department of Experimental and Clinical Medicine, University of Florence, Florence, Italy; 5 European Network for the Study of Adrenal Tumors (ENS@T) Center of Excellence, Florence, 50139 Italy; 6 Emergency Surgery, Careggi University Hospital, Florence, 59100 Italy

**Keywords:** Endocrine, Oncology, Pheochromocytoma, Paraganglioma, Anesthesia

## Abstract

**Background:**

Pheochromocytoma (PHEO) and paraganglioma (PGL) are rare neuroendocrine tumors characterized by hemodynamic instability, caused by the paroxysmal release of catecholamines. Patients may develop cardiovascular complications in the perioperative phase due to the massive release of catecholamines, particularly during anesthetic induction and surgical manipulation of the tumor. The aim of this retrospective study was to evaluate the risk factors involved in perioperative hemodynamic instability in patients who underwent surgery for chromaffin tumors.

**Methods:**

Forty patients (median age 55 [36.50–64.50]) undergone surgery for PHEO/abdominal PGL from January 2011 to December 2016 at the AOU Careggi (Florence, Italy) were retrospectively evaluated. Systolic, diastolic, and mean blood pressure were considered at baseline and during surgery. Patients with blood pressure steadily < 140/90 mmHg before surgery were considered “adequately prepared”. A preoperative therapy with doxazosin, a selective alpha-1 blocker, was started in all patients for at least 14 days prior to the surgery. The presence of hemodynamic instability was reported.

**Results:**

Comparing males and females, a significant difference in doxazosin daily dose (*p* = 0.018), systolic blood pressure (*p* = 0.048), and in the proportion of adequately prepared patients (*p* = 0.031) emerged. A positive correlation between preoperative daily dose of doxazosin, tumor size (*B* = 0.60, *p* < 0.001), and urinary normetanephrine levels (*B* = 0.64, *p* < 0.001) was also observed. Hemodynamic instability occurred in 30.0% of patients. The absence of adequate preparation (*p* = 0.012) before surgery, urinary normetanephrine levels (NMNur *p* = 0.039), and surgery time (minutes) (*p* = 0.021) resulted as risk factors of hemodynamic instability in our series. The use of intraoperative drugs was higher in patients with hemodynamic instability (*p* < 0.001). A pre-surgical SBP level of > 133 mmHg (OR = 6 CI95% 1.37–26.20, *p* = 0.017) and an intraoperative SBP and MBP levels of > 127 mmHg (OR = 28.80 CI95% 2.23–371.0, *p* = 0.010) and > 90 mmHg (OR = 18.90 CI95% 1.82–196.0, *p* = 0.014), respectively, were identified as effective thresholds to recognize patients at higher risk of HI.

**Conclusions:**

A preoperative therapy with alpha-blockers is useful, but not sufficient to avoid surgical risks. Patients with higher pre-surgical levels of NMNur, pre-surgical SBP > 133 mmHg, and/or intraoperative SBP > 127 mmHg and MBP > 90 mmHg, should be carefully monitored. A multidisciplinary approach is indispensable to optimize the management of PHEOs/abdominal PGLs in order to reduce surgical complications.

## Background

Pheochromocytoma (PHEO) and paraganglioma (PGL) are rare neural-crest derived tumors. Their incidence, comparable in males and females, is 0.8 cases/100,000/year with a peak between the 3rd and the 5th decade [1, 2]. To date, surgery represents the treatment of choice [1].

Up to 70% of PHEOs and PGLs (PPGLs) are caused by germline or somatic genetic variants in one of the susceptibility gene. According to the transcription profile, two main clusters are reported: Cluster 1 includes genes characterized by the activation of the pseudohypoxia signaling (*SDHA, SDHB, SDHC, SDHD, SDHAF2, VHL, FH, EPAS1*), and Cluster 2 includes genes related to the activation of kinase signaling (*NF1, RET, TMEM127, MAX, HRAS*) [3].

PPGLs are characterized by elevated hemodynamic instability (HI) and cardiovascular mortality due to catecholamines (CA) release [4]. In particular, this risk increases during surgery, especially during anesthetic induction and surgical manipulation of the tumor when a massive release of CA can cause hypertensive crises [5]. In the last years, different retrospective studies did not report any differences in mortality and in intraoperative hemodynamic parameters comparing patients treated or not before surgical procedures with alfa-blockers [6]. Anyway, current guidelines recommend treating all patients affected by PPGLs with an adequate alpha-adrenergic receptor blockade starting 7–14 days before surgery [1]. Alpha-adrenergic antagonists are able to neutralize the cardiovascular effects of CA, reduce peripheral vascular resistances and blood pressure levels, expand circulating blood volume and antagonize alpha receptors downregulation [5].

To date, there are no randomized controlled studies establishing the optimal target blood pressure (BP) before surgery but a blood pressure less than 130/80 mmHg while seated and a systolic blood pressure (SBP) higher than 90 mmHg while standing are often considered acceptable [1]. Nevertheless, the optimal target should be evaluated in each patient in agreement with age and cardiological comorbidities [1].

Regarding normotensive patients with PPGL (40% of cases), there are conflicting data on the opportunity of a preoperative alpha-adrenergic blockade [7]. However, potential catastrophic effects due to massive release of CA during surgery support an adequate preparation also in these patients [1, 8]. Also regarding the definition of HI conflicting data are present [9–11].

The aim of this retrospective study was to evaluate the risk factors involved in perioperative HI in patients who underwent surgery for chromaffin tumors, in order to optimize the management of these rare tumors reducing the surgical complications.

## Materials and methods

### Population of the study

We retrospectively collected clinical presurgical data of 40 consecutive patients, median age at diagnosis of 55 years [36.50–64.50], who underwent surgery for PPGLs at the AOU Careggi (Florence, Italy) between January 2011 and December 2016. Thirty-four patients were affected by a PHEO and 6 by abdominal PGL. Thirty-six patients experienced a laparoscopic surgery (33 PHEOs, 3 PGLs) otherwise in 4 a laparotomic approach (1 PHEO, 3 PGLs) was preferred. The laparoscopic surgery was reserved to patients with smaller lesions (37.86 ± 16.41 mm *vs* 51.25 ± 6.29 mm). Genetic analysis was available for 34 patients. Twelve patients (35.30%) presented a mutation in one of the susceptibility genes for PPGLs (5 *RET*, 3 *SDHB*, 2 *NF1*, 1 *PHD2*, 1 *VHL*). We obtained hemodynamic parameters—such as systolic blood pressure (SBP), diastolic blood pressure (DBP), mean blood pressure [MBP = DBP + 1/3 (SBP-DBP)] and heart rate (HR)—at baseline and during the surgical procedure. The presence of cardiological comorbidities (CC), as heart failure and/or arrhythmias, and history of diabetes mellitus were considered. The ASA Physical Status Classification System [12] was used to predict perioperative risk. The diagnosis of PPGLs was based on biochemical parameters (urinary metanephrine and normetanephrine), radiological imaging (CT and/or MRI and/or ^131^I-MIBG scintigraphy), and then confirmed by histology.

### Pre-surgical medical therapy

All patients were treated before surgery with doxazosin according to therapeutic / diagnostic process of adrenal tumors approved at AOU Careggi (PT/903/41). Doxazosin was started for at least 14 days before surgery with a starting dose of 2 mg per day. The daily dose was adjusted until maximum dose of 16 mg per day. In patients which did not achieve adequate blood pressure levels, therapy with calcium antagonists was added (amlodipine 5–10 mg/day or nifedipine 30–60 mg/day). In patients with tachycardia, beta blockers such as propranolol (20–40 mg/twice a day) or atenolol (25–50 mg/day) were prescribed. A high-sodium diet and fluid intake was recommended for several days (4–5 days) before the surgical procedure.

### Adequate preparation and hemodynamic instability

Patients with steadily BP < 140/90 mmHg before surgery were considered “adequately prepared” (AP) by the pharmacological therapy. Surgery was performed on patients under general anesthesia. During surgical sessions, hemodynamic parameters were continuosly monitored. Also the occurrence of tachycardia (HR > 100 bpm) and bradycardia (< 60 bpm) during surgery was reported. Accordingly, during each surgical session, HI was defined by the presence of at least two of the following parameters: SBP > 150 mmHg and/or SBP < 80 mmHg and/or MBP < 60 mmHg, as previously reported [11, 13–15]. The use of antihypertensive drugs during surgery was assessed and the correlation between clinical, biochemical, and hemodynamic parameters was analyzed.

### Statistical analysis

After Kolmogorov–Smirnov test, normal variables were expressed as mean ± standard deviation (SD) and non-normal variables were expressed as median and interquartile range [IQR]. Student’s test for parametric variables and Mann–Whitney test for non-parametric variables were used for comparison between groups. Linear regressions were performed for the assessment of univariate relationships between two continuous variables and these associations were further verified in a multivariate analysis adjusting for age at diagnosis and gender. Considering the pre-surgical and during surgery SBP, DBP, and MBP, receiver operating characteristic (ROC) analysis was performed, using the HI as a readout, to find the relative blood pressure thresholds, sensitivity, and specificity. Thereafter, according to the sample size, these thresholds and other significant predictors found at the univariate analysis were simultaneously introduced in a stepwise regression model based on the Akaike Information Criterion (AIC), to find the best-fitting model. All the variables found significant at univariate analysis were entered into a multivariate linear regression analysis. A *p *value of < 0.05 was considered significant. Statistical analysis was performed using SPSS version 27.0 (SPSS Inc., Chicago), R version 4.1.2 (2021–11–01), GraphPad Prism version 9.0.0 for Windows, GraphPad Software, San Diego, CA, USA, www.graphpad.com.

## Results

Table 1 shows patients’ characteristics, considering the overall population, and according to the gender (i.e., males and females). Fifteen patients (37.5%) presented pre-surgical blood pressure less than 120/80 mmHg. A selective alpha-1 blocker (doxazosin) was used at personalized doses in all patients for at least 14 days before surgery, as a unique therapy or in combination with others antihypersive drugs. In addition to doxazosin, beta blockers were administered in nine patients, calcium antagonists were prescribed in two cases and only one patient was treated with beta blockers and calcium antagonists.

**Table 1:** Characteristics of patients

Characteristics	Overall population (*n* = 40)	Males (*n* = 18)	Females (*n* = 22)	*P* value
Age at diagnosis	55 [36.50–64.50]	54.40 [41.00–66.00]	55.00 [33.00–63.75]	0.596
Body mass index (BMI) kg/m^2^	23.85 ± 3.90	23.73 ± 3.09	23.95 ± 4.52	0.427
PHEO/abdominal PGL, *n* (%)	35 (87.50)/5 (12.50)	14 (77.8)/4 (22.2)	20 (90.9)/2 (9.1)	0.381
Mutated patients, *n* (%)	12/34 (35.30) *RET* 5 (41.7) *SDHB* 3 (25) *NF1* 2 (16.7) *PHD2* 1 (16.7) *VHL* 1 (8.3)	4/18 (22.2) *RET* 2 (11.1) *SDHB* 1 (5.6) *NF1* 1 (5.6) *PHD2* 0 (0.0) *VHL* 0 (0.0)	8/22 (36.4) *RET* 3 (13.6) *SDHB* 2 (9.1) *NF1* 1 (4.5) *PHD2* 1 (4.5) *VHL* 1 (4.5)	0.853
Surgical approach	Laparoscopic 36 (90)	Laparoscopic 15 (83.3)	Laparoscopic 21 (95.5)	0.310
	Laparotomic 4 (10)	Laparotomic 3 (16.7)	Laparotomic 1 (4.5)	
Secretory phenotype	Noradrenergic 18 (45.00)	Noradrenergic 8 (44.4)	Noradrenergic 10 (45.5)	
Noradrenergic/adrenergic,* n (%)*	Adrenergic 22 (55.00)	Adrenergic 10 (55.6)	Adrenergic 12 (54.5)	1.000
Secretory phenotype				
Urinary metanephrine (µg/24 h)	513 [132.25–1435.25]	610.5 [174.75–1960]	418 [119.75–1307.75]	0.652
Urinary normetanephrine (µg/24 h)	1256.5 [712–4439]	1705.50 [850.75–4990.25]	1073 [568.75–3917]	0.229
Tumor size (mm)	39.20 ± 16.17	41.94 ± 20.67	36.95 ± 11.30	0.367
Preoperative antihypertensive therapy				0.101
Alpha blockers, *n (%)*	25 (62.50)	9 (50.0)	17 (77.3)	
Alpha blockers + others, *n (%)*	15 (37.50)	9 (50.0)	5 (22.7)	
Mean doxazosin daily dose (mg)	4.90 ± 2.80	6.06 ± 3.60	3.95 ± 1.58	**0.018**
Pre-surgical systolic blood pressure (SBP) mmHg	127.15 ± 16.24	131.94 ± 16.73	123.23 ± 15.08	**0.048**
Pre-surgical dyastolic blood pressure DBP mmHg	76.03 ± 11.16	76.11 ± 11.44	75.95 ± 11.19	0.483
Cardiological comorbidities, *n* (%)	6 (15.00)	5 (27.8)	1 (4.5)	0.073
Diabetes mellitus, *n* (%)	1 (2.50)	0 (0.0)	1 (4.5)	1.000
Adequately prepared (AP), *n* (%)	31 (77.5)	11 (61.1)	20 (90.9)	**0.031**
ASA Physical Status Classification System, *n* (%)	I 14 (35)	I 6 (33.3)	I 8 (36.4)	0.844
	II 26 (65)	II 12 (66.7)	II 14 (63.6)	

In the *P* value column the bold font indicates a significant value < 0.05

A laparoscopic approach was performed in 90% of cases, median surgery time was 110 min [90.00–148.75]. The anesthetic induction was obtained by the use of propofol and/or midazolam, tracrium, and fentanyl and maintained with remifentanil.

**Fig. 1:**
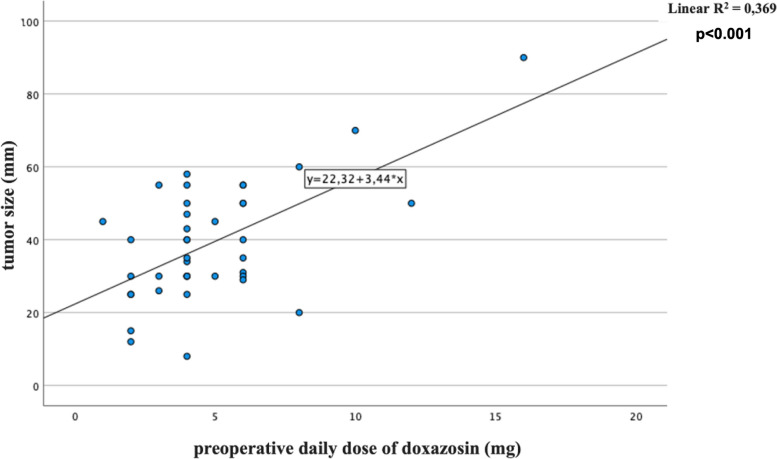
Correlation between preoperative daily dose of doxazosin (mg/day) and tumor size (mm)

**Fig. 2:**
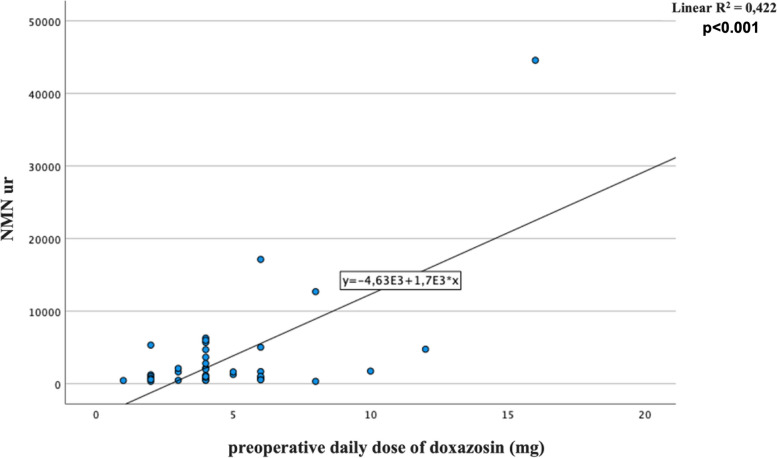
Correlation between preoperative daily dose of doxazosin (mg/day) and levels of urinary normetanephrine (NMNur)

**Fig. 3:**
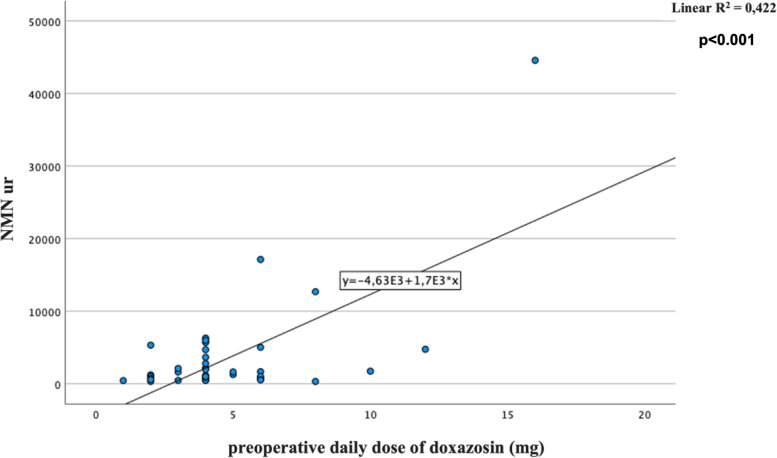
Correlation between tumor size and urinary normetanephrine levels (NMNur)

No significant surgical complications occurred, except in one patient, who required a blood transfusion for an intraoperative bleeding. According to presurgical cardiovascular parameters, an AP was achieved in 31 out of 40 patients (77.50%). Comparing AP and no AP patients we found a significantly difference only considering pre-surgical SBP (*p* = 0.003). Comparing males and females, significant differences emerged according to the doxazosin daily dose (6.06 ± 3.60 *vs* 3.95 ± 1.58, *p* = 0.018), the pre-surgical SBP (131.94 ± 16.73 *vs* 123.23 ± 15.08, *p* = 0.048) and the number of adequately prepared patients (11 (61.1%) *vs* 20 (90.9%), *p* = 0.031). To rule out possible confounders, the doxazosin doses were also corrected for the body mass index (BMI) and pre-surgical SBP, confirming an independent and significant gender difference in this factor (*p* = 0.041 ancd *p* = 0.047, respectively). At univariate analysis, we found a significant correlation between preoperative doxazosin daily dose and tumor size as well as urinary normetanephrine (NMNur) (*B* = 0.6 and 0.64, respectively, all *p* < 0.001) (Figs. 1 and 2). Associations were confirmed at multivariate analysis after introducing age at diagnosis of PPGLs and gender as confounders (*B* = 0.62, *p* < 0.001 and *B* = 0.67, *p* < 0.001, respectively).A significant correlation also emerged between tumor size and NMNur (*B* = 0.63, *p* < 0.001) (Fig. 3). The correlation was confirmed at multivariate analysis after adjusting for the aforementioned covariates (*B* 0.61, *p* < 0.001). Bradycardia and tachycardia occurred in 7.50% and in 2.50% of patients, respectively.

HI occurred in 30.00% (*n* = 12) of patients. The characteristics of patients without (group I) and with (group II) HI are shown in Table 2. Briefly, as expected, the incidence of HI was greater in “non-AP” (*p* = 0.012). Furthermore, the use of intraoperative medications was significatively higher in the group II (7 (25%) *vs* 10 (83.3%), *p* < 0.001), who also presented higher levels of pre-surgical NMNur (959.0 [559.50–2641.25] *vs* 2047 [1059.25–5601.50] *vs p* = 0.039).

**Table 2:** Characteristics of patients without (group I) and with (group II) hemodynamic instability (HI)

Characteristics	Group I (*n* = 28)	Group II (*n* = 12)	*P* value
Male, *n* (%)	12 (42.9)	6 (50.0)	0.471
Age at diagnosis PHEO/PGL	50.61 ± 16.20	51.25 ± 22.18	0.919
Extra-adrenal localization (%)	4/28 (14.3)	2/12 (16.7)	0.595
Tumor size (mm)	37.00 ± 16.22	44.33 ± 15.51	0.192
Cluster			0.896
1	4 (16.00)	1 (10.00)	
2	5 (20.00)	2 (20.00)	
wt	16 (64.00)	7 (70.00)	
Mean doxazosin daily dose (mg/die)	4.68 ± 2.75	5.42 ± 3.15	0.461
Urinary metanephrine (µg/24 h)	519 [125.25–1283.25]	513 [219–2421.25]	0.497
Urinary normetanephrine (µg/24 h)	959.0 [559.50–2641.25]	2047 [1059.25–5601.50]	**0.039**
Pre-surgical SBP (mmHg)	123.9 ± 14.8	134.7 ± 17.5	0.0051
Pre-surgical DBP (mmHg)	76.46 ± 11.20	75.00 ± 11.48	0.918
Pre-surgical MBP (mmHg)	92.32 ± 11.54	94.83 v 11.34	0.668
Adequate preparation (%)	25 (89.30)	6 (50.00)	**0.012**
Preoperative antihypertensive therapy			0.311
Alpha blockers,* n (%)*	19 (67.9%)	6 (50%)	
Alpha blockers + others, *n (%)*	9 (32%)	6 (50%)	
ASA Physical Status Classification System, *n* (%)	I 9 (32.1)	I 5 (41.7)	0.568
	II 19 (67.9)	II 7 (58.3)	
Laparoscopic approach (%)	26 (92.90)	10 (83.30)	0.346
Surgery time (min)	107.50 [81.25–138.75]	130.00 [102.50–206.25]	**0.021**
Intraoperative drugs (%)	7 (25.00)	10 (83.30)	** < 0.001**
Nitrates	0 (0%)	2 (16.7%)	
Alpha-blockers	2 (7.1%)	4 (33.3%)	
Beta-blockers	2 (7.1%)	0 (0%)	
Alpha blockers plus sympathomimetic drugs	1 (3.6%)	0 (0%)	
Nitrates plus alpha blockers	1 (3.6%)	0 (0%)	
Nitrates plus alpha-blockers plus beta-blockers	0 (0%)	1 (8.3%)	
Beta-blockers plus sympathomimetic drugs	0 (0%)	2 (16.7%)	
Beta-blockers plus nitrates	1 (3.6%)	0 (0%)	
Alpha-blockers plus beta-blockers plus sympathomimetic drugs	0 (0%)	1 (8.3%)	
Intraoperative SBP (mmHg)	117.77 ± 7.52	141.48 ± 14.47	** < 0.001**
Intraoperative DBP (mmHg)	68.33 ± 7.17	82.14 ± 10.56	** < 0.001**
Intraoperative MBP (mmHg)	84.81 ± 6.04	101.92 ± 10.76	** < 0.001**
Cardiological comorbidities (%)	2 (7.10)	4 (33.30)	**0.055**

*PHEO* pheochromocytoma, *PGL* paraganglioma, *Adequate preparation* blood pression < 140/90 mmHg before surgery, *Intraoperative drugs* drugs administered to control hemodynamic parameters; *Cardiological comorbidities* heart failure and/or arrhythmias

In the *P* value column the bold font indicates a significant value < 0.05

**Fig. 4:**
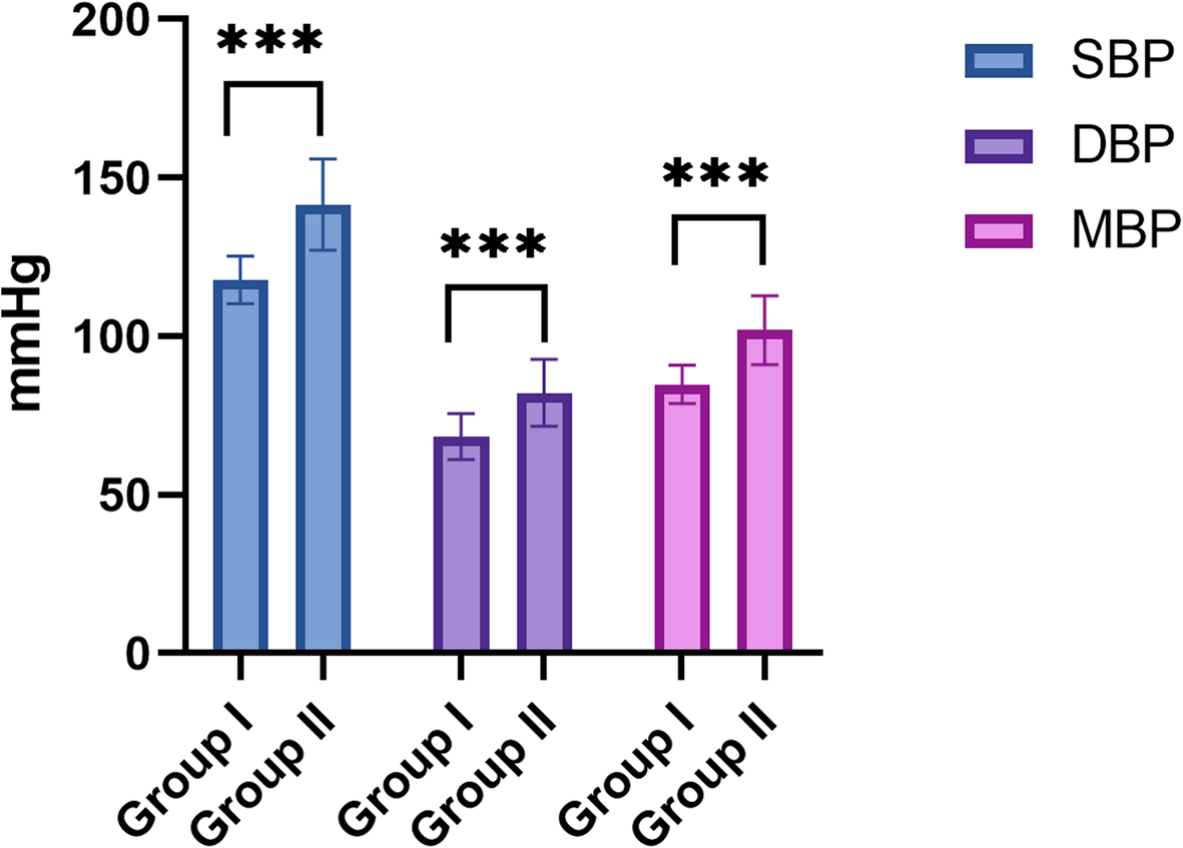
comparison between median SBP, DBP and MBP considering group I and group II. Notes SBP = systolic blood pressure, DBP = diastolic blood pressure, MBP = mean blood pressure. Group I without HI, Group II with HI, *** *p* < 0.0001

Considering the hemodynamic parameters, only pre-surgical SBP levels approached the significance between group I and II (123.9 ± 14.8 vs 134.7 ± 17.5, *p* = 0.0051). On the other hand, intraoperative blood pressure levels resulted significantly different between the two groups, as shown in Fig. 4. Of note, Group I presented lower SBP (117.77 ± 7.52 *vs* 141.48 ± 14.47 mmHg, *p* < 0.001), lower DBP (68.33 ± 7.17 *vs* 82.14 ± 10.56 mmHg, *p* < 0.001), and lower MBP (84.81 ± 6.04 *vs* 101.92 ± 10.76, *p* < 0.001).

### Prognostic factors

**Fig. 5:**
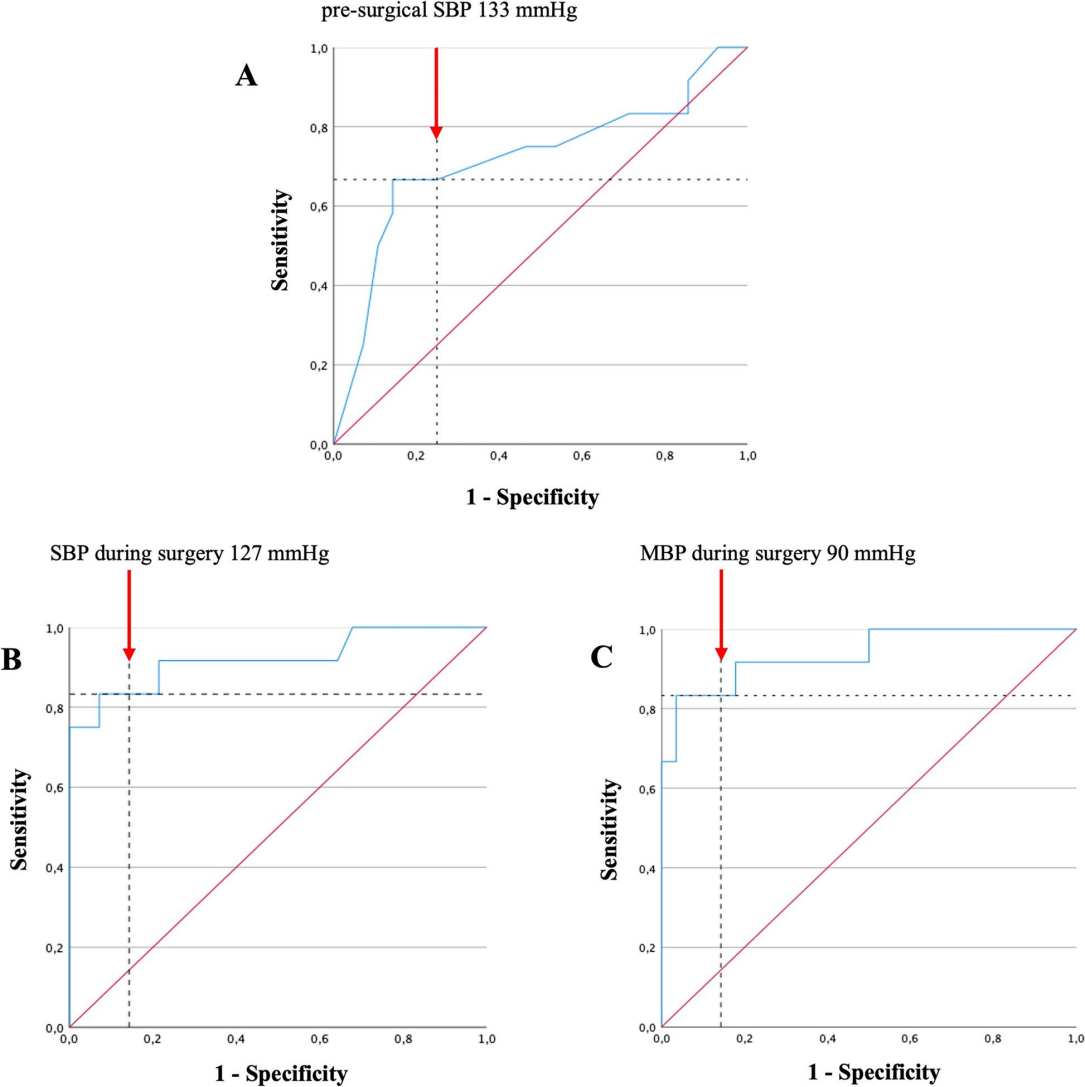
**A** ROC curve analysis considering pre-surgical SBP according to HI. The AUC was 0.719 (95% CI 0.524–0.914), *p* = 0.030. A value higher than 133 mmHg shows a sensitivity of 66.7% and a specificity of 75%. **B** ROC curve analysis considering SBP during surgery according to HI. The AUC was 0.921 (95% CI 0.801–1.00), *p* < 0.0001. A value higher than 127 mmHg shows a sensitivity of 83.3% and a specificity of 85.7%. **C** ROC curve analysis considering MBP during surgery according to HI. The AUC was 0.937 (95% CI 0.851–1.00), *p* < 0.0001. A value higher than 90 mmHg shows a sensitivity of 83.3% and a specificity of 85.7%. Notes: SBP systolic blood pressure, MBP blood pressure, HI hemodynamic instability

In order to find the best pre-surgical blood pressure thresholds, a ROC curve analysis was performed, considering the SBP, DBP, and MBP values and using the HI as a readout: a pre-surgical SBP > 133 mmHg showed a sensitivity of 66.7% and a specificity of 75% (AUC = 0.719, CI95% = 0.524–0.914, *p* = 0.030) (Fig. 5A). We did not find a statistically significant thresholds for pre-surgical DBP and MBP.

In addition, according to the sample-size, two separate stepwise regression analysis by AIC were perfomed, using HI as dependent variable and the most significant presurgical and intraoperative factors. The former included the presurgical SBP threshold, along with the NMNur values, and ASA Physical Status Classification System. The best-fitting model showed that only the presurgical SBP independently influences the hemodynamic outcome. In particular, a pre-surgical SBP higher than 133 mmHg significantly increased the risk of HI (OR = 6 (CI95% 1.37–26.20, *p* = 0.017) during surgery. The same model was confirmed also after including sex as covariate along with NMNur values and ASA Physical Status Classification System (not shown).

Secondly, we investigated the relationship among the intraoperative blood pressure thresholds and the HI. A ROC curve analysis was performed, considering the intraoperative SBP, DBP, and MBP values and using the HI as a readout. All three parameters revealed very high accuracy in predicting the final outcome (*p* < 0.0001 all): a SBP > 127 mmHg showed a sensitivity of 83.3% and a specificity of 85.7% (AUC = 0.921, CI95% 0.811–1.000) (Fig. 5B); a DBP > 78 mmHg showed a sensitivity of 75% and a specificity of 89.3% (AUC = 0.876, CI95%:0.768–0.985); while a MBP > 90 mmHg showed a sensitivity of 83.3% and a specificity of 85.7% (AUC = 0.937, CI95% 0.851–1.000) (Fig. 5C).

The second stepwise regression analysis by AIC was perfomed, using HI as dependent variable and including the blood pressure thresholds, along with the NMNur values (Table 3). We found that a level of SBP and MBP higher than the reported thresholds (127 and 90 mmHg, respectively) significantly increased the risk of HI (SBP OR = 28.80 CI95% 2.23–371.0, *p* = 0.010 and MBP OR = 18.90 CI95% 1.82–196.0, *p* = 0.014) during surgery.

**Table 3:** Intraoperative best-fitting model, from a stepwise regression analysis by AIC using the Hemodynamic Instability as dependent variable, and after introducing the blood pressure thresholds during surgery, and urinary normetanephrine values

95% Confidence interval				
	Odd ratio	Lower	Upper	*P* value
SBP > 127 mmHg	28.80	2.23	371.0	**0.010**
MBP > 90 mmHg	18.90	1.82	196.0	**0.014**
NMN ur	1.00	1.00	1.00	0.082

*AIC* Akaike Information Criterion, *SBP* Systolic blood pressure, *MBP* Mean blood pressure, *NMN* Urinary normetanephrine levels

In the *P* value column the bold font indicates a significant value < 0.05

## Discussion

PPGLs are rare neuroendocrine tumors characterized by either production or release of CA. Surgery is the treatment of choice and, currently, minimally invasive techniques (laparoscopy or robotic surgery) are used to reduce perioperative complications. The improvement in anesthetic management has been useful to minimize this risk. However, the anesthetic induction, the creation of pneumoperitoneum, as well as the mobilization of the tumor during surgery, may induce a massive release of CA leading to an increase of perioperative mortality and morbidity [10, 16–20].

Our retrospective study aimed to identify the risk factors for intraoperative HI in patients undergone surgery for PPGLs. Forty patients (34 PHEOs and 6 abdominal PGLs) were included. As expected, a group of patients (15/40, 37.5%) presented normal pre-surgical blood pressure (less than 120/80 mmHg). In fact, in particular patients with adrenal lesions may have normal blood pressure with potential hypertensive crises. Despite this, a selective alpha-1 blocker (doxazosin) was used in all patients for at least 14 days before surgery, as a unique therapy or in combination with others antihypertensive drugs. We considered AP patients with steadily BP < 140/90 mmHg. Comparing AP and non AP patients only a significantly difference in pre-surgical SBP emerged, while pre-surgical DPM and MBP were comparable. As it was reported in a recent meta-analysis, there are different definitions of intraoperative HI in the literature [11]. In our opinion, the alteration of only one hemodynamic parameter is not enough to establish the occurence of HI. In fact, we defined the presence of HI if at least two of the below parameters were present in our series: SBP > 150 mmHg, SBP < 80 mmHg and MBP < 60 mmHg. We did not consider the heart rate (HR) in the definition of HI taking into account that it was used only in few studies. In our population HI occurred in 30.00% of cases.

The tumor size was not identified as a risk factor for HI. Conflicting data were reported in the literature regarding its role: some authors reported higher incidence of HI in patients with larger tumors [10, 16, 21–23], while others did not find a correlation between tumor size and HI [18].

NMNur level was demonstrated as a risk factor for HI in our patients. Our data proved the evidence of a positive correlation between tumor size and NMN levels as previously demonstrated [15]. The relationship between tumor size and NMNur could be explained by a greater CA releasing ability of larger tumors [24].

Differently from literature data [25], the surgical technique was not an influent factor on HI, but it should be noted that a laparotomic approach was performed only in 4 patients (two in each group).

The presence of cardiological comorbidities (CC) resulted as a risk factor of HI development in our population, even if at the limit of statistical significance. Negative effects of CA on the cardiovascular system, such as endothelial dysfunction, vascular remodeling [26–28], and cardiac hypertrophy induction [29] are well known. Differently, we didn’t find any significantly differences considering the ASA Physical Status Classification System. In fact, controversial data are present regarding its use as risk predictor [9].

As expected, patients who presented intraoperative HI needed more drug administration compared to the group without HI. No significant differences emerged in doxazosin daily dose between the group with and without HI; therefore, it was not possible to identify a “threshold dose” able to predict a reduction of the risk of HI development.

We did not found any correlation between HI and genetic profile in our population. Differently, Pang et al. recently demonstrated that Cluster 2 was an independent risk factor of intraoperative HI, although 49.8% of patients undergone genetic analysis belonged to Cluster 2 *vs* 21.3% of patients that presented a variant linked to Cluster 1 [30]. We have to highlight that patients who experienced HI were significantly less adequately prepared before surgery compared to the group of patients in which HI did not occur (50.00% vs 89.3%, *p* = 0.012). Therefore, our data show that the achievement of AP prior to the surgery permits to reduce the occurrence of HI and improves perioperative outcomes. In addition, patients with higher pre-surgical levels of NMNur and with longer time of surgery should be carefully monitored because of a possible greater risk of HI.

We also identified a treshold for pre-surgical SBP, and SBP and MBP during surgery to recognize patients at higher risk of intraoperative HI. Firstly, we defined HI by choosing the most appropriate term among those present in the literature [11]. Secondly, ROC curve analysis was used to better evaluate the blood pressure trend before and throughout the procedure.

Nevertheless, our result also demonstrate that an adequate pharmacological preparation does not guarantee the absence of significant variations in intraoperative blood pressure parameters requiring treatment. This finding is not surprising as presurgical treatment with alpha-blockers is aimed at reducing catecholamine-induced vasoconstriction and re-expand plasma volume as well as reducing the occurrence of hypertensive crises before surgery but during surgery other factors such as increased intraabdominal pressure necessary for laparoscopy or the surgical manipulation of the tumor, may occur leading to unpredictable cardiovascular effects. For this reason, even for patients adequately prepared, an expert anesthesiologist is required in assisting them during the surgical procedure.

It is worth mentioning that in our study all patients were prepared with doxazozin, a selective, competitive alpha-blocker which may be displaced by tumor CA released in high quantity during surgery. This event does not apply for phenoxybenzamine, a non-selective, non-competitive alpha-blocker, not worldwide available, which cannot be displaced by the receptor, and which a metanalysis has been demonstrated more effective than doxazosin in avoiding intraoperative hemodynamic instability [31].

The strength of this study is represented by the homogeneity of the pre-surgical therapeutic approach employed and by the attempt to identify potential risk factors involved in perioperative HI, an important issue that is not extensively studied to date. Secondly, to our knowledge, for the first time we identified a pre-surgical SBP cut-off and an intraoperative SBP and MBP cut-off to recognize patients at higher risk of intraoperative HI. In particular, the pre-surgical SBP cut-off could assist clinicians preparing patients for surgery. The main limitations of our study were the small size of the recruited population, the retrospective design, and the lack of a control group. Furthermore, we did not conduct a sub-analysis considering the drugs used for anesthesia and during surgery. Howewer, there are no dedicated guidelines for anesthesia or intraoperative treatment in patients affected by PHEO/PGL [9].

PPGLs surgery still represents a challenge due to the associated risk of HI. Ad adequate medical preparation with alpha-blockers is useful but not sufficient to limit perioperative risks. A multidisciplinary approach involving different experts (endocrinologist, surgeon, anesthesiologist) is advisable to optimize the management of a complex and rare disease as PPGLs.

## Data Availability

Data are available from the corresponding author upon reasonable request.

## Abbreviations

PHEO: Pheochromocytoma
PGL: Paraganglioma
PPGL: Pheochromocytoma and paraganglioma
HI: Hemodynamic instability
CA: Catecholamines
SBP: Systolic blood pressure
DBP: Diastolic blood pressure
MBP: Mean blood pressure
HR: Heart rate
CTC: Computed tomography
MRI: Magnetic resonance imaging
^131^I-MIBG: ^131^I-metaiodobenzylguanidine
BP: Blood pressure
AP: Adequately preparated
CC: Cardiological comorbidities
SD: Standard deviation
NMNur: Urinary normetanephrine
COMT: Cathecol-O-methyl transferase

## References

[1] Lenders JW, Duh QY, Eisenhofer G, Gimenez-Roqueplo AP, Grebe SK, Murad MH (2014). Pheochromocytoma and paraganglioma: an endocrine society clinical practice guideline. J Clin Endocrinol Metab.

[2] Chen H, Sippel RS, O'Dorisio MS, Vinik AI, Lloyd RV, Pacak K (2010). The North American Neuroendocrine Tumor Society consensus guideline for the diagnosis and management of neuroendocrine tumors: pheochromocytoma, paraganglioma, and medullary thyroid cancer. Pancreas.

[3] Dahia PL (2014). Pheochromocytoma and paraganglioma pathogenesis: learning from genetic heterogeneity. Nat Rev Cancer.

[4] Lenders JW, Eisenhofer G, Mannelli M, Pacak K (2005). Phaeochromocytoma. Lancet.

[5] Mannelli M (2006). Management and treatment of pheochromocytomas and paragangliomas. Ann N Y Acad Sci.

[6] Schimmack S, Kaiser J, Probst P, Kalkum E, Diener MK, Strobel O (2020). Meta-analysis of α-blockade versus no blockade before adrenalectomy for phaeochromocytoma. Br J Surg.

[7] Isaacs M, Lee P (2017). Preoperative alpha-blockade in phaeochromocytoma and paraganglioma: is it always necessary?. Clin Endocrinol (Oxf).

[8] Agarwal A, Gupta S, Mishra AK, Singh N, Mishra SK (2005). Normotensive pheochromocytoma: institutional experience. World J Surg.

[9] Araujo-Castro M, Pascual-Corrales E, Nattero Chavez L, Martínez Lorca A, Alonso-Gordoa T, Molina-Cerrillo J (2021). Protocol for presurgical and anesthetic management of pheochromocytomas and sympathetic paragangliomas: a multidisciplinary approach. J Endocrinol Invest.

[10] Brunaud L, Nguyen-Thi PL, Mirallie E, Raffaelli M, Vriens M, Theveniaud PE (2016). Predictive factors for postoperative morbidity after laparoscopic adrenalectomy for pheochromocytoma: a multicenter retrospective analysis in 225 patients. Surg Endosc.

[11] Bihain F, Nomine-Criqui C, Guerci P, Gasman S, Klein M, Brunaud L (2022). Management of patients with treatment of pheochromocytoma: a critical appraisal. Cancers (Basel)..

[12] Horvath B, Kloesel B, Todd MM, Cole DJ, Prielipp RC (2021). The evolution, current value, and future of the American society of anesthesiologists physical status classification system. Anesthesiology.

[13] Gaujoux S, Bonnet S, Lentschener C, Thillois JM, Duboc D, Bertherat J (2016). Preoperative risk factors of hemodynamic instability during laparoscopic adrenalectomy for pheochromocytoma. Surg Endosc.

[14] Tauzin-Fin P, Sesay M, Gosse P, Ballanger P (2004). Effects of perioperative alpha1 block on haemodynamic control during laparoscopic surgery for phaeochromocytoma. Br J Anaesth.

[15] Chang RY, Lang BH, Wong KP, Lo CY (2014). High pre-operative urinary norepinephrine is an independent determinant of peri-operative hemodynamic instability in unilateral pheochromocytoma/paraganglioma removal. World J Surg.

[16] Kiernan CM, Solórzano CC (2016). Pheochromocytoma and paraganglioma: diagnosis, genetics, and treatment. Surg Oncol Clin N Am.

[17] Kercher KW, Novitsky YW, Park A, Matthews BD, Litwin DE, Heniford BT (2005). Laparoscopic curative resection of pheochromocytomas. Ann Surg.

[18] Plouin PF, Duclos JM, Soppelsa F, Boublil G, Chatellier G (2001). Factors associated with perioperative morbidity and mortality in patients with pheochromocytoma: analysis of 165 operations at a single center. J Clin Endocrinol Metab.

[19] Joris JL, Hamoir EE, Hartstein GM, Meurisse MR, Hubert BM, Charlier CJ (1999). Hemodynamic changes and catecholamine release during laparoscopic adrenalectomy for pheochromocytoma. Anesth Analg.

[20] Aliyev S, Karabulut K, Agcaoglu O, Wolf K, Mitchell J, Siperstein A (2013). Robotic versus laparoscopic adrenalectomy for pheochromocytoma. Ann Surg Oncol.

[21] Bruynzeel H, Feelders RA, Groenland TH, van den Meiracker AH, van Eijck CH, Lange JF (2010). Risk factors for hemodynamic instability during surgery for pheochromocytoma. J Clin Endocrinol Metab.

[22] Kinney MA, Warner ME, vanHeerden JA, Horlocker TT, Young WF, Schroeder DR (2000). Perianesthetic risks and outcomes of pheochromocytoma and paraganglioma resection. Anesth Analg.

[23] Livingstone M, Duttchen K, Thompson J, Sunderani Z, Hawboldt G, Sarah Rose M (2015). Hemodynamic stability during pheochromocytoma resection: lessons learned over the last two decades. Ann Surg Oncol.

[24] Eisenhofer G, Deutschbein T, Constantinescu G, Langton K, Pamporaki C, Calsina B (2020). Plasma metanephrines and prospective prediction of tumor location, size and mutation type in patients with pheochromocytoma and paraganglioma. Clin Chem Lab Med.

[25] Bai S, Yao Z, Zhu X, Li Z, Jiang Y, Wang R (2019). Comparison of transperitoneal laparoscopic versus open adrenalectomy for large pheochromocytoma: a retrospective propensity score-matched cohort study. Int J Surg.

[26] Higashi Y, Sasaki S, Nakagawa K, Kimura M, Noma K, Matsuura H (2002). Excess norepinephrine impairs both endothelium-dependent and -independent vasodilation in patients with pheochromocytoma. Hypertension.

[27] Head RJ (1991). Hypernoradrenergic innervation and vascular smooth muscle hyperplastic change. Blood Vessels.

[28] Dao HH, Lemay J, de Champlain J, deBlois D, Moreau P (2001). Norepinephrine-induced aortic hyperplasia and extracellular matrix deposition are endothelin-dependent. J Hypertens.

[29] Galetta F, Franzoni F, Bernini G, Poupak F, Carpi A, Cini G (2010). Cardiovascular complications in patients with pheochromocytoma: a mini-review. Biomed Pharmacother.

[30] Pang Y, Li M, Jiang J, Chen X, Fu Y, Wang C (2022). Impact of body composition and genotype on haemodynamics during surgery for pheochromocytoma and paraganglioma. J Cachexia Sarcopenia Muscle.

[31] Zawadzka K, Więckowski K, Małczak P, Wysocki M, Major P, Pędziwiatr M (2021). Selective vs non-selective alpha-blockade prior to adrenalectomy for pheochromocytoma: systematic review and meta-analysis. Eur J Endocrinol.

