# Correction: Hemodynamic parameters in patients undergoing surgery for pheochromocytoma/paraganglioma: a retrospective study

**DOI:** 10.1186/s12957-023-03181-9

**Published:** 2023-09-19

**Authors:** Giuseppina De Filpo, Gabriele Parenti, Clotilde Sparano, Giulia Rastrelli, Elena Rapizzi, Serena Martinelli, Francesca Amore, Benedetta Badii, Prosperi Paolo, Tonino Ercolino, Massimo Mannelli, Mario Maggi, Letizia Canu

**Affiliations:** 1 Department of Experimental and Clinical Biomedical Sciences “Mario Serio”, University of Florence, Florence, Italy; 2 Endocrinologic Unit, Azienda Ospedaliera Universitaria Careggi, Florence, Italy; 3 Sexual Medicine and Andrology Unit Department of Experimental and Clinical Biomedical Sciences “Mario Serio”, University of Florence, Florence, Italy; 4 Department of Experimental and Clinical Medicine, University of Florence, Florence, Italy; 5 European Network for the Study of Adrenal Tumors (ENS@T) Center of Excellence, Florence, 50139 Italy; 6 Emergency Surgery, Careggi University Hospital, Florence, 59100 Italy

**Correction: World J Surg Onc 21, 192 (2023)**

**https://doi.org/10.1186/s12957-023-03072-z**

Following publication of the original article [1], the author reported that the given names and family names were switched.

The original article has been updated.
